# Porcine model of a complicated skin and soft tissue infection caused by *Pseudomonas aeruginosa*

**DOI:** 10.17221/25/2024-VETMED

**Published:** 2024-06-27

**Authors:** Bretislav Lipovy, Lukas Vacek, Dominika Polastik Kleknerova, Edita Jeklova, Lenka Liskova, Jakub Holoubek, Dominika Matyskova, Filip Ruzicka

**Affiliations:** ^1^Department of Burns and Plastic Surgery, Institution Shared with University Hospital Brno, Faculty of Medicine, Masaryk University, Brno, Czech Republic; ^2^Advanced Biomaterials Group, Central European Institute of Technology, Brno University of Technology, Brno, Czech Republic; ^3^Department of Microbiology, St. Anne’s University Hospital and Faculty of Medicine, Masaryk University, Brno, Czech Republic; ^4^Clinical Immunology and Immunology of Infectious Diseases, Veterinary Research Institute, Brno, Czech Republic; ^5^Department of Histology and Embryology, Faculty of Medicine, Masaryk University, Brno, Czech Republic

**Keywords:** ESKAPE pathogen, model development, pig, wound

## Abstract

*Pseudomonas aeruginosa* poses a significant threat to both immunocompetent and immunocompromised individuals, often resulting in life-threatening infections. With increasing antimicrobial resistance, novel therapeutic strategies are urgently needed. Although animal models are crucial for preclinical studies, limited data are available for porcine models, more specifically for *P. aeruginosa* complicated skin and soft tissue infections (cSSTIs). This study presents a novel porcine model inducing and sustaining cSSTI for 14 days. Six pigs (120 wounds) were used for the development of infections, and within this group, two pigs (40 wounds) were used to evaluate the progression of the cSSTI infection. The model demonstrated bacterial loads of more than 10^7^ CFU/gram of tissue or higher. The cSSTI fully developed within three days and remained well above these levels until day 14 post-infection. Due to the immunocompetence of this model, all the immunological processes associated with the response to the presence of infection and the wound healing process are preserved.

Due to the increasing incidence of infectious complications and the dramatic rise in antimicrobial resistance, the World Health Organization has included *Pseudomonas aeruginosa* in the so-called ESKAPE list (*Enterococcus faecium*, *Staphylococcus aureus*, *Klebsiella pneumoniae*, *Acinetobacter baumannii*, *Pseudomonas aeruginosa* and *Enterobacter* sp.), i.e., pathogens against which new antimicrobial agents and strategies are urgently needed (Mulani et al. 2019; Mancuso et al. 2021). For the implementation of new antimicrobial biomaterials and additives in human medicine, it is necessary to evaluate their potential not only under *in vitro* conditions but also in the *in vivo* context. Animals provide a comprehensive model for testing the topical administration of antimicrobial biomaterials and their additives to simulate a real clinical situation, complicated skin and soft tissue infection (cSSTIs) (Dai et al. 2011). In this publication, we present our strategy for introducing cSSTI in the pig (*Sus scrofa f. domestica*).

## MATERIAL AND METHODS

### Porcine model

The animal care protocol for this experiment followed the Czech Guidelines for Animal Experimentation and was approved by the Central Commission for Animal Welfare of the Ministry of Agriculture of the Czech Republic (Serial No. MZe 2181). A total of six pigs were used throughout the experiment. Six pigs (120 wounds) were used for the development of infection, and within this group, two pigs (40 wounds) were used to further evaluate the progression of the cSSTI infection in an animal model. Female hybrid Large White (50%) × Landrace (50%) pigs (Bioprodukt Knapovec a.s., Usti nad Orlici, Czech Republic), aged 15 weeks, with an average body weight of 75 ± 5 kg were used for the study. The pigs were housed individually in enriched environment pens in the accredited barrier-type animal facilities of the Veterinary Research Institute, Brno. The pigs were fed a standard commercial diet (De Heus, Marefy, Czech Republic) twice a day *ad libitum* and had unlimited access to drinking water.

### Development of the complicated skin and soft tissue infection

All the surgical procedures were performed under general anaesthesia. The premedication and analgesia during the surgery were provided by the subcutaneous administration of butorphanol (0.1 mg/kg of body weight; b.w.; Richter Pharma AG, Wels, Austria), a synthetic opioid agonist-antagonist analgesic. Anaesthesia was induced with a combination of tiletamine/zolazepam (2 mg/kg of b.w.; Virbac, Carros, France), ketamine (2 mg/kg of b.w.; Bioveta, Ivanovice na Hane, Czech Republic), and xylazine (2 mg/kg of b.w.; Bioveta, Ivanovice na Hane, Czech Republic) administered intramuscularly. General anaesthesia was then maintained by an intravenous propofol administration (8–15 mg/kg of b.w.; Fresenius Kabi GmbH, Graz, Austria).

**Figure 1:**
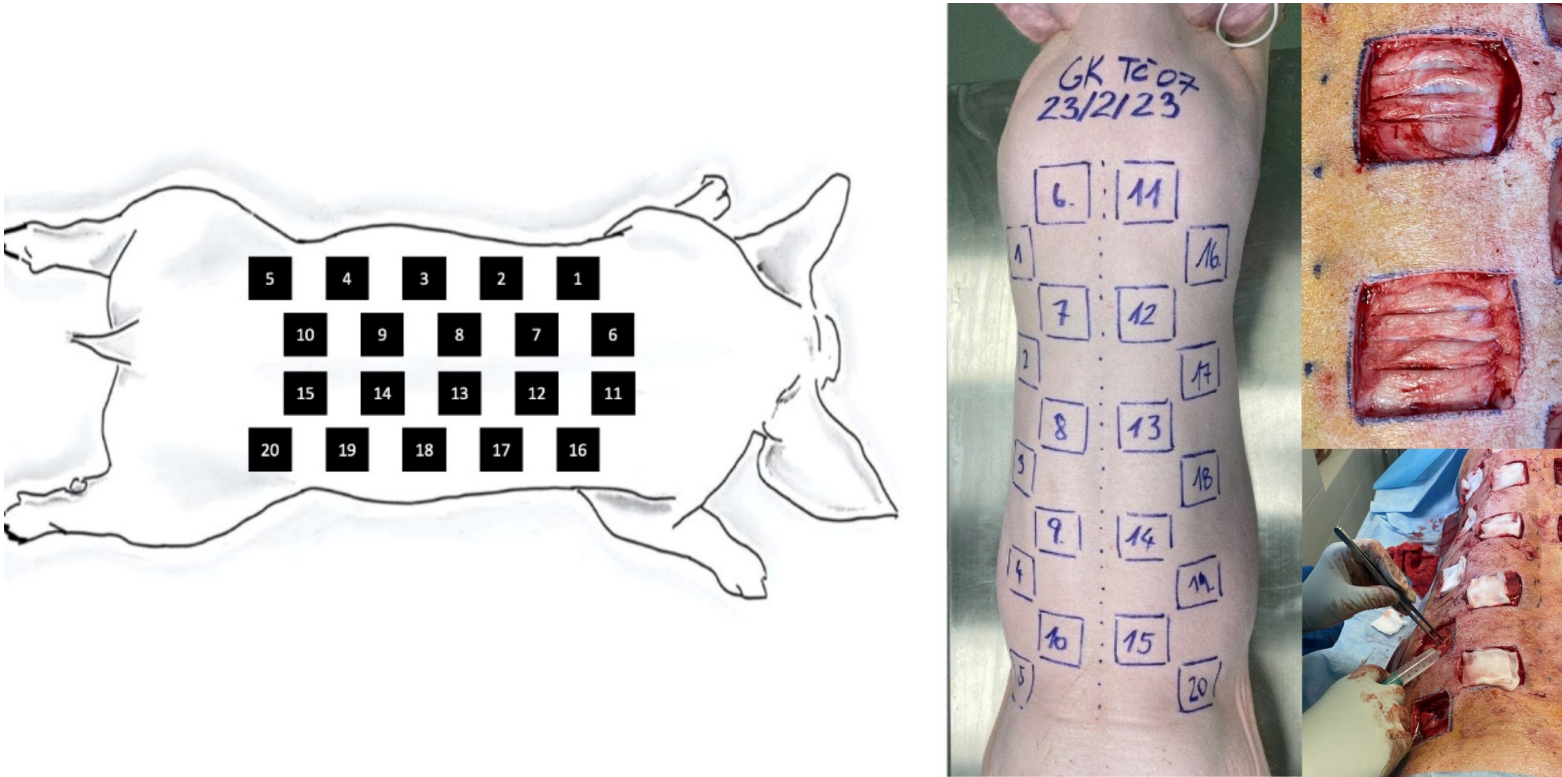
The position of skin defects on the back of the pig in the animal models Twenty skin defects, ten on each side of the spine, were performed with fascial incisions and inoculation of *P. aeruginosa*

Full-thickness skin defects were surgically performed on the backs of the pigs after antisepsis (povidone-iodine alcoholic solution, followed by 2% peracetic acid). Skin excisions were performed in strictly defined areas of 5 × 5 cm. Twenty skin defects were performed in each pig, ten on each side of the vertebral column (Figure 1). Fascial incisions were performed inside the defects to introduce a complicated deep skin and soft tissue infection (fasciitis and myonecrosis) and maintain the infection for the entire duration of the experiment.

The multi-resistant *P. aeruginosa* clinical strain FF2 (producing VIM carbapenemase) was used for the wound infection development. Five (5) ml of bacterial inoculum (concentration approx. 10^9^ CFU/ml) was diffusely applied onto the sterile gauzes, which individually covered the surgically induced skin defects. Once the wounds were infected, the occlusive dressing was fixed on the pigs’ backs. Immediately after surgery, a second analgesic drug, meloxicam (0.1 mg/kg b.w.), was administered subcutaneously and then continued at a dose of 0.1 mg/kg b.w. once a day for the next three days.

### Microbiological assessment of the infection development

Microbiological and histopathological samples were taken on days 4, 7, 11 and 14 post-infection to assess the level of bacterial load in the wounds during the study. Preoperative antibiotic prophylaxis was used to reduce the risk of contaminating agents affecting the induced infection. Specifically, ceftazidime pentahydrate (2 g; Fresenius Kabi GmbH, Graz, Austria) and shotapen (combination of benzathine benzylpenicillin 10 mg/kg, procaine benzylpenicillin 10 mg/kg, and dihydrostreptomycin sulphate 20.5 mg/kg; Virbac, Carros, France) were administered prior to sampling. The *P. aeruginosa* strain used in this study is resistant to these antibiotics according to the European Committee on Antimicrobial Susceptibility Testing (EUCAST) methods and interpretation criteria (The European Committee on Antimicrobial Susceptibility Testing 2023).

The bacterial load on the wound surface was assessed using the filter paper imprint technique (Chovanec et al. 2014), and the intra-tissue bacterial load was assessed using a microbiological evaluation of the tissue biopsy samples.

#### FILTER PAPER IMPRINT TECHNIQUE

The filter paper imprint technique allows for the identification and semi-quantitative assessment of the number of microorganisms present on the wound surface. In brief, sterile Whatman Grade 4 cellulose filter papers (size 5 × 5 cm, Sigma-Aldrich, St. Louis, MO, USA) serve as a transfer vehicle between the wound surface and the surface of the culture medium. The filter papers were applied and pressed against the wound surface and left there for one minute to absorb the wound exudate. They were then transferred onto the surface of the appropriate culture media, in this case, on the blood agar (containing Columbia agar base with 5% sheep blood; Oxoid, Hampshire, UK) and on the selective culture medium (blood agar containing 10% NaCl; Oxoid, Hampshire, UK). The culture media were then incubated at 37 °C for 24 h and visually evaluated.

The results were scored as follows: score “0” = 0 CFU/25 cm^2^, score “1” = 1–10 CFU/25 cm^2^, score “2” = 11–100 CFU/25 cm^2^, score “3” = 101–1 000 CFU/25 cm^2^, score “4” > 1 000 CFU/25 cm^2^.

#### TISSUE BIOPSY SAMPLING

The bacterial load within the tissue was assessed by processing the tissue biopsy samples. Tissue samples were taken using an 8 mm biopsy punch (Medplus, Havirov, Czech Republic), placed in 2 ml of sterile phosphate-buffered saline (PBS), and homogenised (Stomacher^®^ 80 Biomaster, Seward, UK). Serial ten-fold dilutions of the homogenate were plated onto blood agar and blood agar supplemented with 10% NaCl. Plates were incubated at 37 °C for 24 h, after which the colony-forming unit (CFU) counts were enumerated, and the CFU per gram of the tissue was calculated. Bacterial colonies were subsequently analysed by Matrix-Assisted Laser Desorption/Ionisation Time-of-Flight Mass Spectrometry (MALDI-TOF MS; Bruker, Fallanden, Switzerland) to verify the presence of the expected bacterial species in the infected wounds and to detect any contamination of the samples. In addition, pulsed-field gel electrophoresis was used as a molecular typing tool to ensure that the isolated and the original bacterial strains were identical.

### Histopathological assessment of the infection development

The tissue samples for the histopathological examination were fixed in a 10% formaldehyde solution for 24 hours. The material was processed according to standardised protocols using an ETP PLUS tissue samples processor (Intelsint Srl, Villarbasse, Italy). All the samples were embedded into paraffin blocks using a TES 99 Paraffin Embedding Center (Medite Medical, Burgdorf Germany). Samples were sectioned using a Thermo Scientific Microm HM 325 Rotary Microtome (Thermo Fisher Scientific, Wien, Austria). Serial slices were mounted on standard glass slides (Knittel Glass Gmbh, Braunschweig, Germany) and stained by Haematoxylin-eosin and Gram staining (Bamed, Litvinovice, Czech Republic). The prepared slides were examined for signs of inflammation, fibrosis, vascular dilatation, and the number and localisation of bacterial cells.

### Visual assessment of the infection development

In addition to the microbiological and histopathological assessment, the visual appearance and the local signs of the wound infection, such as purulent discharge, redness, or induration, were assessed.

### Statistical analysis

To statistically evaluate the infection development, the bacterial loads (expressed as log CFU/gram tissue or a scoring value corresponding to CFU/25 cm^2^ of wound surface area) in samples were compared. An analysis of variance (ANOVA) test was used to compare bacterial loads in six pigs on day 4. A paired *t*-test was used to compare the results on days 4 vs 7, 4 vs 11, and 4 vs 14 post-infection to determine whether the bacterial load in the wounds had changed significantly. The significance level used for the tests was α = 0.05. Statistica v14 software (TIBCO Software, Santa Clara, CA, USA) was used for the statistical analysis.

## RESULTS

To assess the bacterial load in the wounds during the experiment, samples were taken on days 4, 7, 11, and 14 post-infection. The bacterial load on the wound surface was assessed using the filter paper imprint technique and a visual assessment. The tissue load was assessed by microbiological evaluation of the tissue biopsy samples and histopathological evaluation.

### Assessment of the infection development

The data obtained from the microbiological evaluation of the imprint samples showed a mean scoring of 3.95 ± 0.25 (corresponding to log CFU/25 cm^2^ of wound surface area) on day 4 post-infection in all six animals tested.

**Figure 2:**
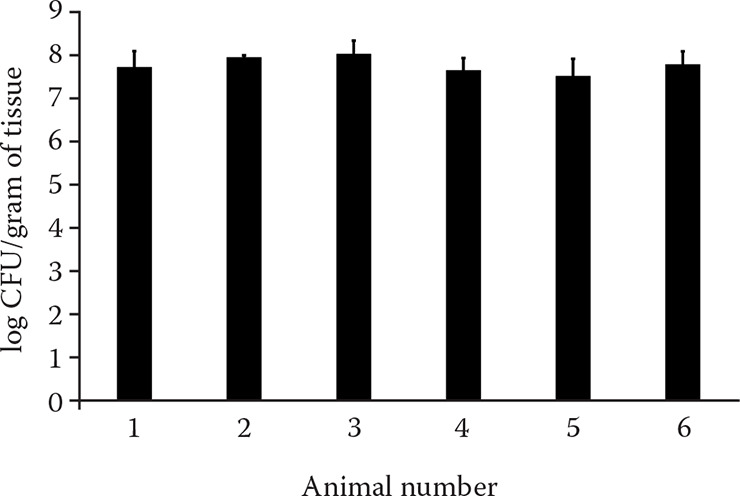
Infection development after four days after infection Six pigs were infected (120 wounds) with a mean bacterial load of 7.82 ± 0.35 log CFU/gram of tissue. The bacterial load was not significantly different among the tested animals, *P* > 0.05

The tissue biopsy data samples showed a mean bacterial load of 7.82 ± 0.35 log CFU/gram of tissue on day 4 post-infection in all the tested animals. It was statistically confirmed that the bacterial load was not significantly different among the tested animals, *P* > 0.05 (Figure 2).

**Figure 3:**
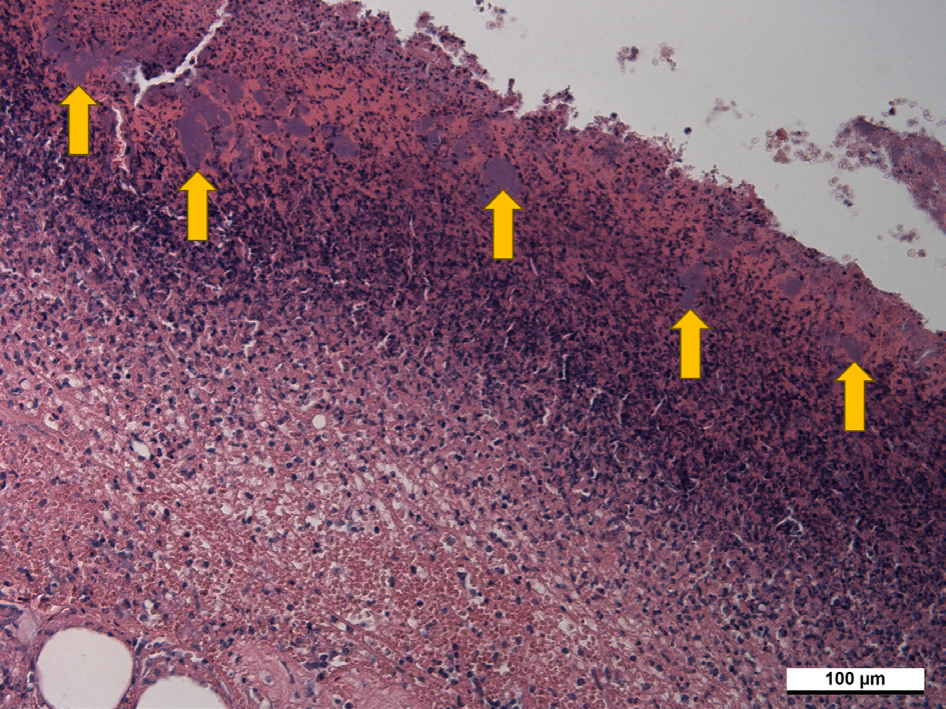
Histopathological evaluation (haematoxylin-eosin staining) of the complicated SSTI development The samples were characterised by significant tissue infiltration by inflammatory cells, with a predominance of polymorphonuclear cells and macrophages. The samples also showed extensive areas of bacterial cells within the wound tissue (yellow arrows) and on its surface (× 200 magnification)

The histopathological evaluation further confirmed the full development of cSSTI. The samples were characterised by significant tissue infiltration by inflammatory cells, with a predominance of polymorphonuclear cells and macrophages. The samples showed extensive areas of bacterial cells within the wound tissue and on its surface (Figure 3).

### Assessment of the infection progress within 14 days post-infection

Two pigs (40 wounds) were used to further evaluate the progression of the cSSTI infection in the animal model.

**Figure 4:**
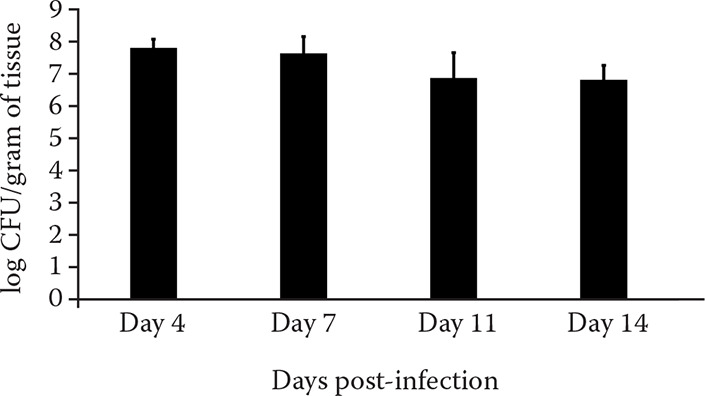
Infection progression within 14 days post-infection Two pigs were infected (40 wounds) with mean bacterial loads of 7.89 ± 0.27, 7.73 ± 0.52, 6.96 ± 0.79, and 6.90 ± 0.45 log CFU/gram of tissue on day 4, 7, 11, and 14 post-infection. It was statistically confirmed that the bacterial load on day 7 was not significantly different from day 4 (*P* = 0.501). However, there was a statistically significant decrease in the bacterial load on days 11 and 14 compared to day 4, *P* = 0.023 and *P* = 0.001, respectively

The data from the microbiological evaluation of the imprint samples showed mean score values of 3.85 ± 0.43, 3.98 ± 0.16, 3.80 ± 0.41, and 3.55 ± 0.55 (corresponding to log CFU/25 cm^2^ of wound surface area) on days 4, 7, 11, and 14 post-infection, respectively. The tissue biopsy data showed mean bacterial loads of 7.89 ± 0.27, 7.73 ± 0.52, 6.96 ± 0.79, and 6.90 ± 0.45 log CFU/gram of tissue on days 4, 7, 11, and 14 post-infection, respectively. It was statistically confirmed that the bacterial load on day 7 was not significantly different from that on day 4 (*P* = 0.501). In contrast, there was a statistically significant decrease in the bacterial load on days 11 and 14 compared to day 4, *P* = 0.023 and *P* = 0.001, respectively (Figure 4).

The histopathological evaluation further confirmed the progression of the cSSTI infection. The specimens were characterised by significant tissue infiltration by inflammatory cells, with a predominance of polymorphonuclear cells and macrophages accompanied by apparent vasodilatation. Hyperaemia related to the granulation process was also observed.

In addition, the samples showed an increased presence of inflammatory infiltrate, which was also observed in the deeper layers as the infection progressed. The samples showed extensive areas of bacterial cells within the wound tissue and on its surface.

### Visual assessment of the infection progress within 14 days post-infection

**Figure 5:**
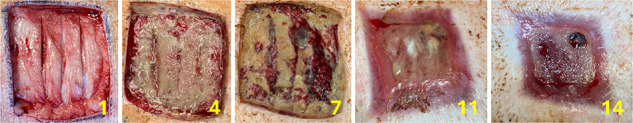
The animal’s wounds showed local signs of inflammation, with purulent discharge remaining until the end of the experiment From day 11, local signs of inflammation receded, with evidence of re-epithelisation and wound contraction

In addition to the microbiological and histopathological assessment, the visual appearance of the wounds was compared throughout the experiment. The wounds of the animals showed local signs of inflammation, with purulent discharge remaining until the end of the experiment. From day 11, local signs of inflammation receded, with evidence of re-epithelisation and wound contraction (Figure 5).

## DISCUSSION

*P. aeruginosa* causes acute and chronic infectious complications in immunocompetent and immunocompromised patients, often resulting in life-threatening infections (Reynolds and Kollef 2021; Spernovasilis et al. 2021). In particular, cSSTI in burn patients is of great concern due to the immunocompromise associated with thermal trauma, massive loss of skin integrity, and the presence of necrotic tissue, which provides an excellent growth environment for many pathogens, including opportunistic agents (Azzopardi et al. 2014; Lipovy et al. 2016).

Due to the dramatic increase in resistance to currently used antimicrobials, efforts are being made to define and develop new approaches and concepts to expand the options for patient therapy.

For these purposes, it is also important to have animal models that, due to their complexity, ensure a real local and systemic response. Several animal models of SSTIs have been described in the literature, most of which involve infections caused by *S. aureus*. Although there are also models for *P. aeruginosa*, many of these refer only to small models – mice or rats for specific localisation of infection. There is limited information in the literature on the implementation of the porcine animal model for *P. aeruginosa* cSSTI (Wood et al. 2023; Dehbashi et al. 2024).

In our model, we were able not only to induce this process, but also to maintain it for 14 days. Although there is a statistically significant reduction in the bacterial load, it is important to note that most studies classify a bacterial load of 10^5^ CFU/gram of tissue or higher as critical for acute or chronic wound infection (Robson and Heggers 1969; Serena et al. 2021; Hurlow and Bowler 2022). In addition, Breidenbach and Trager (1995) demonstrated that a critical bacterial load of ≥ 10^4^ CFU/gram of tissue is required to cause infection in complex extremity wounds. Both criteria were met within three days when the cSSTI fully developed and remained well above these levels until day 14 post-infection.

In this paper, we describe our concept for the development of an infection model in the white pig, in which we have succeeded in repeatedly inducing cSSTI caused by the *P. aeruginosa* strain. Due to the immunocompetence of this model, all the immunological processes associated with the response to the presence of infection and the wound healing process (especially the inflammatory and proliferative phases) are preserved.
